# The Distribution of *Miniature Impala* Elements and *SIX* Genes in the *Fusarium* Genus is Suggestive of Horizontal Gene Transfer

**DOI:** 10.1007/s00239-017-9801-0

**Published:** 2017-07-25

**Authors:** Peter van Dam, Martijn Rep

**Affiliations:** Molecular Plant Pathology, Swammerdam Institute for Life Sciences, University of Amsterdam, Amsterdam, The Netherlands

**Keywords:** Transposable elements, MITE, Inverted repeat, *mimp*, Horizontal gene transfer, Comparative genomics

## Abstract

The *mimp* family of miniature inverted-repeat transposable elements was previously found only in genomes of *Fusarium oxysporum* and is contextually associated with virulence genes in this species. Through extensive comparative analysis of 83 *F. oxysporum* and 52 other *Fusarium* genomes, we uncovered the distribution of different *mimp* families throughout the genus. We show that (i) *mimps* are not exclusive to *F. oxysporum*; (ii) pathogenic isolates generally possess more *mimps* than non-pathogenic strains and (iii) two isolates of *F. hostae* and one *F. proliferatum* isolate display evidence for horizontal transfer of genetic material to or from *F. oxysporum*. Multiple instances of *mimp* elements identical to *F. oxysporum mimps* were encountered in the genomes of these isolates. Moreover, homologs of effector genes *(SIX1, 2, 6, 7, 11* and *FomAVR2)* were discovered here, several with very high (97–100%) pairwise nucleotide sequence identity scores. These three strains were isolated from infected flower bulbs (*Hyacinthus* and *Lilium* spp.). Their ancestors may thus have lived in close proximity to pathogenic strains of *F. oxysporum* f. sp. *hyacinthi* and f. sp. *lilii*. The Fo f. sp. *lycopersici SIX2* effector gene was found to be widely distributed (15/18 isolates) throughout the *F. fujikuroi* species complex, exhibiting a predominantly vertical inheritance pattern. These findings shed light on the potential evolutionary mechanism underlying plant-pathogenicity in *Fusarium* and show that interspecies horizontal gene transfer may have occurred.

**Electronic supplementary material:**

The online version of this article (doi:10.1007/s00239-017-9801-0) contains supplementary material, which is available to authorized users.

## Introduction

Transposable elements (TEs) are DNA sequences that can duplicate or move from one site to another within a genome. Two different TE classes are distinguished based on their transposition intermediate, RNA or DNA. Class I transposons (also called retrotransposons) transpose by transcription into an RNA intermediate and reverse transcription into cDNA before insertion into a new site. Class II TEs (or DNA transposons) on the other hand, transpose through a “cut-and-paste” mechanism. This latter class of TEs is flanked by Terminal Inverted Repeats (TIRs) that facilitate the recognition for DNA excision.

Miniature inverted-repeat transposable elements (MITEs) are short [<500 base pairs (bp)] non-autonomous class II TEs (Bergemann et al. 2008). Their structure resembles defective DNA transposons and they are thought to originate through the deletion of the transposase open reading frame (ORF) between the TIRs (Feschotte and Pritham 2007). Several studies have shown that MITEs can be mobilized by full-length class II TEs (Feschotte et al. 2002; Dufresne et al. 2007; Bergemann et al. 2008).

A MITE called *mimp* (for *m* *iniature* *imp* *ala)* has so far only been described in the filamentous fungus *Fusarium oxysporum* (Fo). Using different approaches, six families of *mimps* have been described in the reference genome *F. oxysporum* f. sp. *lycopersici* 4287, based on the consensus sequence of the inverted repeats (Bergemann et al. 2008). They are characterized by a uniformity in size of about 180–220 bp (Dufresne et al. 2007) and appear to have originated from full-length *impala* elements.

The *impala* family of transposons belongs to the *Tc1/mariner* superfamily of class II transposons (Hua-Van et al. 2001a). This particular TE occurs at a low copy number in the genome of *F. oxysporum*. Although 1–5 copies were detected in most isolates and the TE has been described as an ancient component of the *F. oxysporum* genome (Hua-Van et al. 2001a), they have also been found to be absent in some isolates (Hua-Van et al. 2001b). *Impala* elements contain a single ORF encoding a transposase of 340 amino acids flanked by TIRs of 37 bp (Hua-Van et al. 2001a). They have been shown to be active in at least some strains of *F. oxysporum* (Hua-Van et al. 2001b). Interestingly, the transposase remains functional when transferred into the genome of closely related [*F. moniliforme*, *F. culmorum*, and *F. graminearum* (Hua-Van et al. 2001b; Dufresne et al. 2007; Spanu et al. 2012)] as well as more distantly related fungal species [*Magnaporthe grisea, Aspergillus nidulans, A. fumigatus, Colletotrichum gloeosporioides*, and *Penicillium griseoroseum* (Villalba et al. 2001; Hua-Van et al. 2002; de Queiroz and Daboussi 2003; Firon et al. 2003; Li Destri Nicosia et al. 2004)]. Reinsertion in the genome occurs at TA residues, which are duplicated upon insertion (Dufresne et al. 2007).

Genomes of *F. oxysporum* strains are divided into two compartments. A set of conserved ‘core’ chromosomes is dedicated to housekeeping and vegetative growth, while one or several accessory chromosomes harbor high numbers of TEs and sometimes large segmental duplications. These accessory chromosomes are sometimes directly linked to virulence of the isolate due to the presence of virulence (effector) genes on these chromosomes (Ma et al. 2010). Moreover, they can be horizontally transferred from pathogenic to non-pathogenic strains, thereby conferring the host-specific pathogenicity upon the recipient strain (Ma et al. 2010). In the genome of Fo f. sp. *lycopersici* 4287, 95% of the class II TEs and the majority of *mimps* are present on the accessory chromosomes (Schmidt et al. 2013). Intriguingly, they were found to be significantly overrepresented in the promoter regions (<1500 bp) of known effector genes (named *SIX,* for *S* *ecreted* *I* *n* *X* *ylem*) and other genes that are expressed during plant infection (Schmidt et al. 2013). This association with virulence genes was used to predict novel candidate effectors in the genomes of Fo f. sp. *lycopersici*, Fo f. sp. *melonis*, Fo f. sp. *cucumerinum*, Fo f. sp. *radicis*-*cucumerinum*, and Fo f. sp. *niveum* (Schmidt et al. 2013, 2016; van Dam et al. 2016).

The goal of the current study was to evaluate the distribution of different classes of *mimps* throughout the *Fusarium* genus based on published and novel whole genome sequences. We searched for *mimp*-like elements from the genomes of isolates belonging to six different *Fusarium* species complexes. We find that *mimp* elements are not exclusive to the *F. oxysporum* species complex. Moreover, we find that several *SIX* genes are present in non-*oxysporum Fusarium* strains that also have many *mimps*. Based on these results we explore the possibility of horizontal transfer of genetic material between *Fusarium* species.

## Results

### Identification of *mimps* in Whole Genome Assemblies

Based on the described inverted repeats in Bergemann et al. (2008), we extracted the sequences of *mimps* from all currently available *Fusarium* genome assemblies. These include 83 *F. oxysporum* genomes and 52 genomes from other *Fusarium* species (Supplemental Table S1). We used a consensus sequence generated from the first 16 nucleotides of TIRs of all six previously described *mimp* families to search for the presence of *mimp*-like elements in each of the genomes (‘AGT[GA][GA]G[GAT][TGC]GCAA[TAG]AA’). Stretches of sequence where an instance of this motif was found within 400 bp of another instance in reverse orientation were extracted. In total, 2688 *mimps* were identified. The vast majority (2572) of these were extracted from *F. oxysporum* genomes.

On average, 31 intact *mimps* were found per *F. oxysporum* genome, with numbers ranging from zero in *F. oxysporum* f. sp. *cubense* N2 and two copies in *F. oxysporum* f. sp. *cubense* B2 to 74 in *F. oxysporum* f. sp. *raphani* PHW815. Other isolates where few *mimps* were encountered include *F. oxysporum* f. sp. *nicotianae* and non-plant pathogenic isolates such as MN14 (saprophytic strain isolated from tomato), FOSC3-a (a clinical isolate), and Fo47 (a biocontrol strain) (Supplemental Table S1). These latter three isolates were previously shown to possess relatively few candidate effector genes and also lack copies of *SIX* virulence genes (van Dam et al. 2016). The reason for the absence of a high number of *mimps* and putative virulence genes in these isolates may be attributed to a smaller amount of accessory material.

### The Degree of Genome Assembly Fragmentation Influences Number of *mimps* Identified

**Fig. 1:**
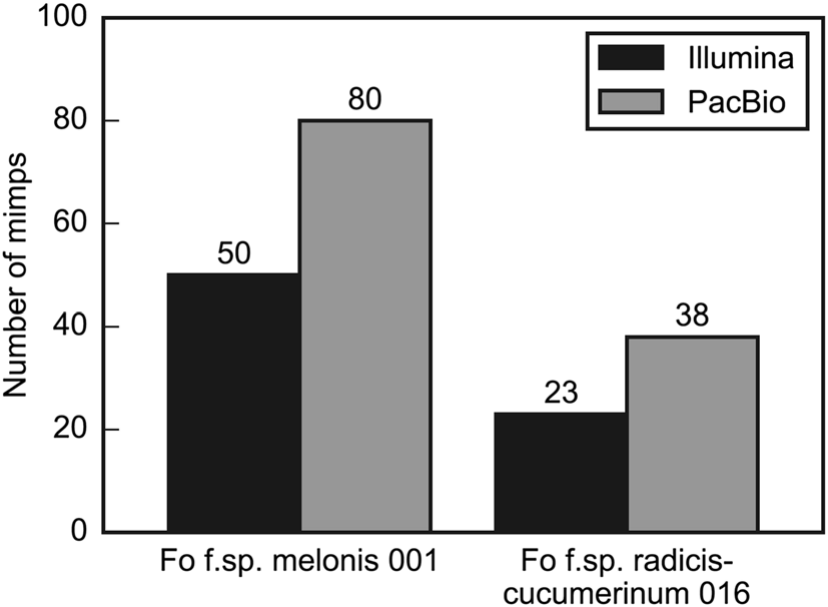
60% (Fo f. sp. *melonis* 001) and 65% (Fo f. sp. *radicis*-*cucumerinum* 016) more intact *mimps* were found in PacBio assemblies compared to Illumina assemblies

*Fusarium* genome assemblies generated from short-read sequence data are typically assembled into hundreds or even thousands of contigs of 500 bp or larger. Especially repeat-rich regions such as the accessory chromosomes of *F. oxysporum*, where most of the *mimps* are typically located (Schmidt et al. 2013; Kang et al. 2014), are highly fragmented. As most of the genomes in our dataset were sequenced with Illumina short read technology, we wondered whether the number of *mimps* was underestimated. We therefore compared the number of *mimps* identified in the Illumina assemblies of two individual *F. oxysporum* isolates to their respective long-read assemblies that were generated from PacBio sequencing data (van Dam et al., accepted). A higher number of intact *mimps* was indeed identified in the PacBio assemblies of *F. oxysporum* f. sp. *melonis* 001 (60% more; 80/50) and *F. oxysporum* f. sp. *radicis*-*cucumerinum* 016 (65% more; 38/23) (Fig. 1).

### Distribution of *mimps* in the *Fusarium* Genus

**Fig. 2:**
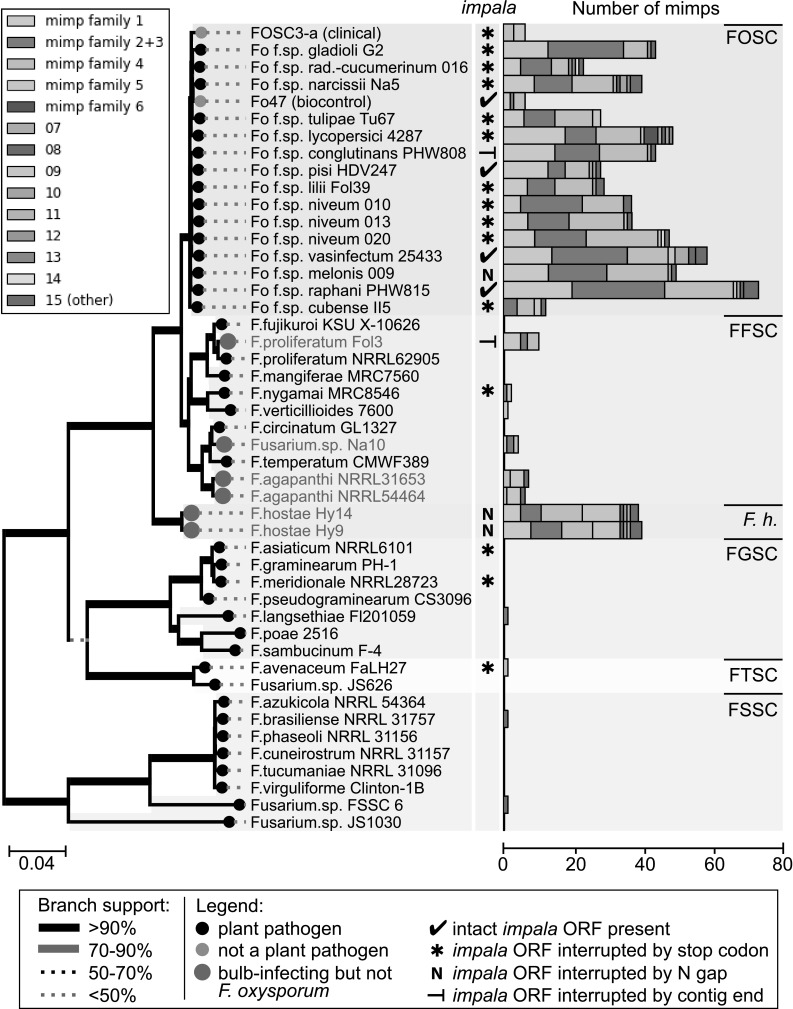
Most *Fusarium* species outside the FOSC lack *mimps* in their genome, but a relatively high number of *mimps* was found in bulb-infecting isolates of *F. hostae* and the FFSC. At least one representative genome was selected per *Fusarium* species. Phylogeny was inferred from a concatenated sequence alignment of three conserved genes: *EF1α, RPB1*, and *RPB2* (partial), using 100 bootstrap replicates. TBLASTN (*e* value < 1e−100) was performed to identify intact *impala* open reading frames in the assemblies. Intact *mimps* were divided into categories and these were plotted next to the phylogenetic tree. In total, the analysis covered six *Fusarium* species complexes (FOSC, *F. oxysporum species complex;* FFSC, *F. fujikuroi species complex;* F. h., *F. hostae;* FGSC, *F. graminearum species complex;* FTSC, *F. tricinctum species complex*; FSSC, *F. solani species complex*)

Although *mimps* have thus far only been described in the *F. oxysporum species complex,* we find that they are not exclusive to this species complex. The genomes of most other *Fusarium* species did show complete absence of *mimps*. However, occurrence of one or a few elements was identified in among others *F. verticillioides, F. proliferatum, F. nygamai*, and *F. avenaceum* (Fig. 2). Two previously sequenced and four de novo sequenced non-*F. oxysporum* genomes stood out in the analysis because they displayed a high number of *mimps*, similar to the numbers found in *F. oxysporum.* These *Fusarium* strains were all isolated from diseased bulb flowers that were affected by bulb rot or leaf and stem spot. *F. proliferatum* Fol3 (isolated from *Lilium*) was earlier identified as *F. oxysporum* (Baayen et al. 1998) but is now reclassified as *F. proliferatum* based on the concatenated sequence of the *EF1alpha, RPB1* and (partial) *RPB2* genes (Fig. 1). *F. hostae* isolates Hy9 and Hy14 were isolated from diseased *Hyacinthus* bulbs (Breeuwsma and De Boer 2004), *F. agapanthi* NRRL31653 and NRRL54464 were isolated from diseased *Agapanthus* plants (African lily) (Edwards et al. 2016) and *Fusarium* sp. Na10 was isolated from diseased *Narcissus* bulbs (Breeuwsma and De Boer 2004).

To identify whether a potentially active intact *impala* was present in the genome, a TBLASTN search was performed using the full-length FOM24 *impala* transposase ORF as a query (Fig. 2). Many *F. oxysporum* isolates showed mutations of the transposase, inducing a premature stop codon in the ORF. However, several isolates such as Fo f. sp. *conglutinans* PHW808, Fo f. sp. *melonis* 009, and Fo47 still possess an intact transposase ORF. Intriguingly, the non-FOSC bulb-infecting isolates *F. hostae* Hy9 and Hy14 as well as *F. proliferatum* Fol3 also contain a largely intact transposase ORF, only interrupted by the end of the contig or by a stretch of ambiguous nucleotides (Ns) caused by contig scaffolding (Fig. 2). This means that an intact *impala* may be present in these isolates. *Fusarium* sp. Na10 and both *F. agapanthi* isolates did not return a significant hit.

Based on reciprocal BLAST hits, all the extracted *mimps* were classified into either of the six previously described families, or novel (unclassified) families and plotted next to a phylogenetic tree of a subselection of the genomes (Fig. 2). The most common *mimp* families are families 1, 2, 3, and 4, which on average make up 86% of the total *F. oxysporum mimp* content. The largest *mimp* family identified in the two *F. agapanthi* isolates (type ‘09’) was not very common in the FOSC. *Mimp* categories in the genomes of bulb-infecting isolates Fol3, Hy9, and Hy14 showed a very similar distribution of families to that found in most *F. oxysporum* isolates. To investigate whether *mimp* elements have been subject to horizontal transfer (HT), we examined their nucleotide sequence in greater detail.

### *Mimp* Elements Identical to *F. oxysporum mimps* Occur Outside the FOSC

**Fig. 3:**
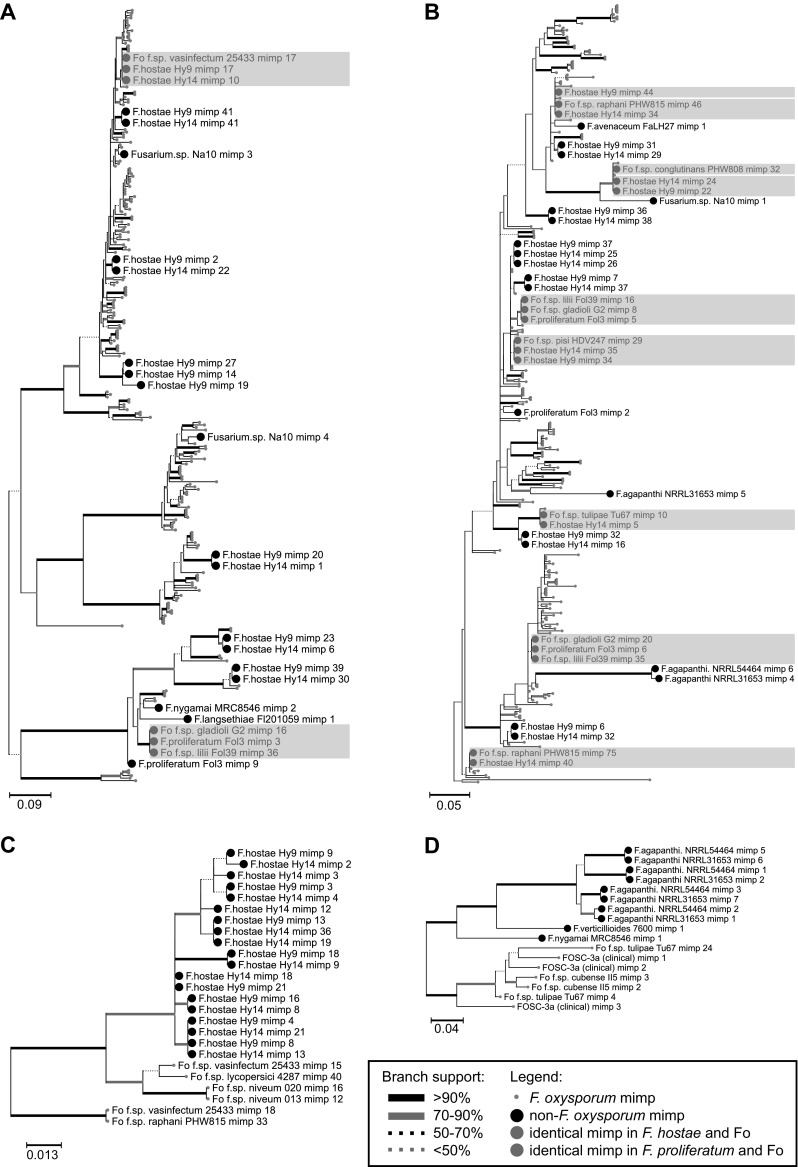
Phylogenies of *mimps* belonging to four different families show the presence of *mimps* in *F. hostae* and *F. proliferatum* Fol3 that are identical to elements found in pathogenic *F. oxysporum* isolates. Sequence alignment was performed on *mimp* sequences extracted from 49 *Fusarium* genomes belonging to **a** *mimp* family 2/3, **b** family 4, **c** family 5, and **d** category ‘09’ with MAFFT and phylogeny was inferred using PhyML with 100 bootstraps. *Mimps* extracted from *F. hostae* and *F. proliferatum* genomes having a 100% identity match in a *F. oxysporum* genome are highlighted in* red* and* blue*, respectively. Node annotations of other *F. oxysporum mimps* have been omitted for clarity

In order to find out whether some of the *mimps* identified in *F. hostae* Hy9/Hy14, *F. proliferatum* Fol3, *Fusarium* sp. Na10, or either of the *F. agapanthi* genomes were (nearly) identical to a copy in one of the 83 *F. oxysporum* genomes, the sequence of each of their *mimps* was compared to all *F. oxysporum mimps* in pairwise comparisons. We found that *F. hostae* Hy9 has five and Hy14 six *mimps* that are 100% identical to copies found in *F. oxysporum* f. sp. *vasinfectum*, *raphani, conglutinans, pisi*, and *tulipae* (Fig. 3a, b). *F. proliferatum* Fol3, isolated from infected lily bulbs, has three *mimps* that are identical to a *F. oxysporum* copy. Interestingly, all three of these elements matched with *mimps* in the genomes of *F. oxysporum* f. sp. *lilii* Fol39 and *F. oxysporum* f. sp. *gladioli* G2, isolates that are also pathogenic to bulb flowers.

The *mimps* belonging to families 2, 3, 4, 5, and category ‘09’ that were identified in the 47 genomes shown in Fig. 2 were aligned per family and visualized in a phylogenetic tree (Fig. 3). This analysis shows that several *F. hostae mimps* and *mimps* extracted from *Fusarium* sp. Na10, *F. proliferatum* Fol3, and *F. nygamai* MRC8546 are present in clades close to *F. oxysporum* elements (Fig. 3a, b), meaning that they are highly similar. Additionally, most *F. agapanthi mimps* (Fig. 3d) and a large number of *F. hostae mimps* (Fig. 3c) are relatively distantly related to *F. oxysporum mimps*, indicating that they might have evolved separately in these species.

### *SIX* Genes Identified in *Fusarium* Species Outside the FOSC

Several sequences with high levels of similarity to a *F. oxysporum SIX* effector gene were identified in Fol3, Hy9, and Hy14. Additionally *FomAVR2*, encoding a small secreted protein with an avirulence function in the Fo f. sp. *melonis*—muskmelon pathosystem, was identified in these strains (Table 1). Both *SIX1* and *FomAVR2* are 100% identical between *F. proliferatum* Fol3 and Fo f. sp. *nicotianae* isolates and several hits in *F. hostae* show a nucleotide similarity of 97% or higher. FOMG_19741 is a gene that was identified as an effector candidate in Fo f. sp. *melonis* based on contextual association with a *mimp* (Schmidt et al. 2016). The reason for including it in Table 1 is the fact that the copy found in Fol3 (isolated from *Lilium* sp.) is highly similar (96.7% identity) to that of Fol39, a Fo f. sp. *lilii* isolate.

**Table 1:** Presence of *F. oxysporum SIX* homologs in *F. proliferatum*, *F. hostae, F. agapanthi* and *Fusarium* sp. isolates indicates possible horizontal transfer of these genes

	Core genes *EF1alpha, RPB1, RPB2*	*SIX1*	*SIX2*	*SIX6*	*SIX7*	*SIX11*	*FomAVR2*	FOMG_19741
*F. proliferatum* Fol3	96.8% on average to Fo	100%^d^ to Fonic 001	73.4% to Focub II5				100% to Fonic 003	96.7% to Folil Fol039
*F. hostae* Hy9	95.5% on average to Fo		86.3% to Focub II5	94.9% to Foniv 019	95.2%^d^ to Folil Fol39^b^	97.6% to Fogla G14^a^	97.4% to Fonic 003	
*F. hostae* Hy14	95.5% on average to Fo		86.3% to Focub II5	94.9% to Foniv 019	95.2%^d^ to Folil Fol39^c^	97.6% to Fogla G14^a^	97.4% to Fonic 003	
*Fusarium* sp. Na10	96.8% on average to Fo		81.4% to Focub II5					
*F. agapanthi* NRRL31653	97.1% on average to Fo		76.6% to Focub II5			96.7% to Fogla G14		
*F. agapanthi* NRRL54464	97.1% on average to Fo		76.5% to Focub II5			96.7% to Fogla G14		

Pairwise identity percentages are based on ClustalO sequence alignments with the best hit of a *F. oxysporum* homolog (Fonic: *F. oxysporum* f. sp. *nicotianae*, Focub: *F. oxysporum* f. sp. *cubense*, Foniv: *F. oxysporum* f. sp. *niveum*, Folil: *F. oxysporum* f. sp. *lilii*, Fogla: *F. oxysporum* f. sp. *gladioli*)

^a^A *mimp* was identified on the same contig as this homolog

^b^Two *mimps* were identified on the same contig as this homolog

^c^Three *mimps* were identified on the same contig as this homolog

^d^This gene is interrupted by multiple stop codons and is probably a pseudogene

**Fig. 4:**
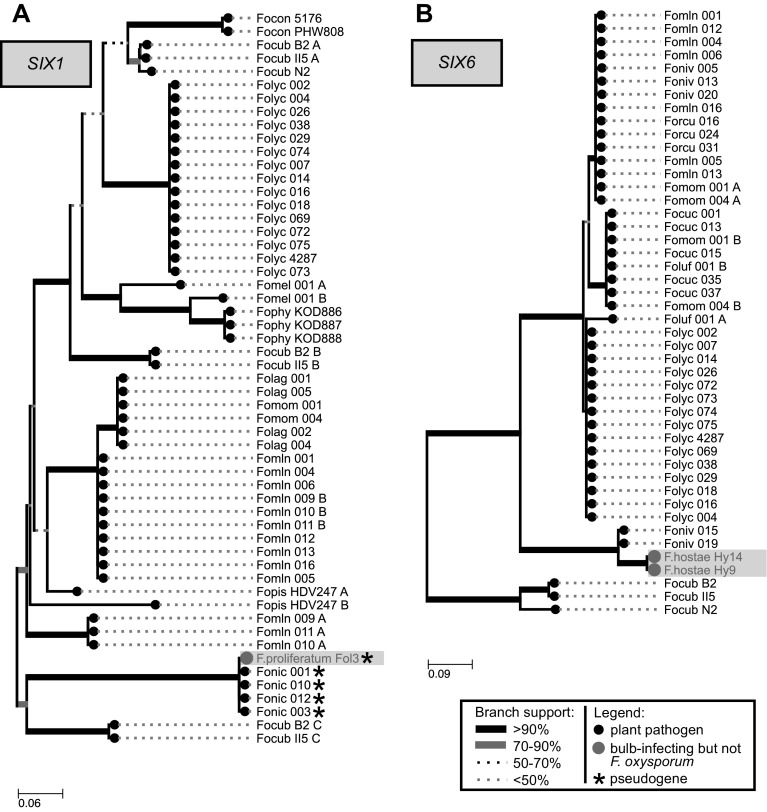
**a** *F. proliferatum* Fol3 has a (pseudogenized) *SIX1* homolog that is identical to Fo f. sp. *nicotianae* SIX1 and **b** *F.hostae SIX6* clusters within the *F. oxysporum* clade, close the *SIX6* gene of Fo f. sp. *niveum 015 and 019*. Clustering of the non-*oxysporum SIX* genes among copies of *F. oxysporum* SIX genes supports the hypothesis of horizontal transfer. Nucleotide sequences were aligned with ClustalO and phylogeny was inferred using PhyML with 100 bootstraps (Fonic: Fo f. sp. *nicotianae*, Focub: Fo f. sp. *cubense*, Foniv: Fo f. sp. *niveum*, Fomln: Fo f. sp. *melonis*, Fopis: Fo f. sp. *pisi*, Folag: Fo f. sp. *lagenariae*; Fomom: Fo f. sp. *momordicae*, Fophy: Fo f. sp. *physali*, Fomel: Fo f. sp. *melongenae*, Focon: Fo f. sp. *conglutinans*, Forcu: Fo f. sp. *radicis*-*cucumerinum*, Focuc: Fo f. sp. *cucumerinum*)

Strikingly, *SIX7* was only discovered in Fo f. sp. *lycopersici* and Fo f. sp. *lilii* Fol39 as an intact ORF. A pseudogenized version of the gene was found in *F. hostae* Hy9/Hy14, as well as Na5 (f. sp. *narcissii*) and G14/G2 (f. sp. *gladioli*); all isolated from diseased flower bulbs. The homologs of *SIX1* (in *F. proliferatum* Fol3) have also undergone mutations resulting in multiple stop codons in its ORFs (Fig. 4a). For the other effector homologs *(SIX2, SIX6, SIX11, FomAVR2*, and FOMG_19741), manual inspection showed that these were intact and potentially active ORFs. The position of *F. proliferatum* Fol3 *SIX1* (pseudogenized, Fig. 4a) and *F. hostae SIX6* (Fig. 4b) in the phylogenetic tree shows them nested within the tree of all *F. oxysporum* homologs.

**Fig. 5:**
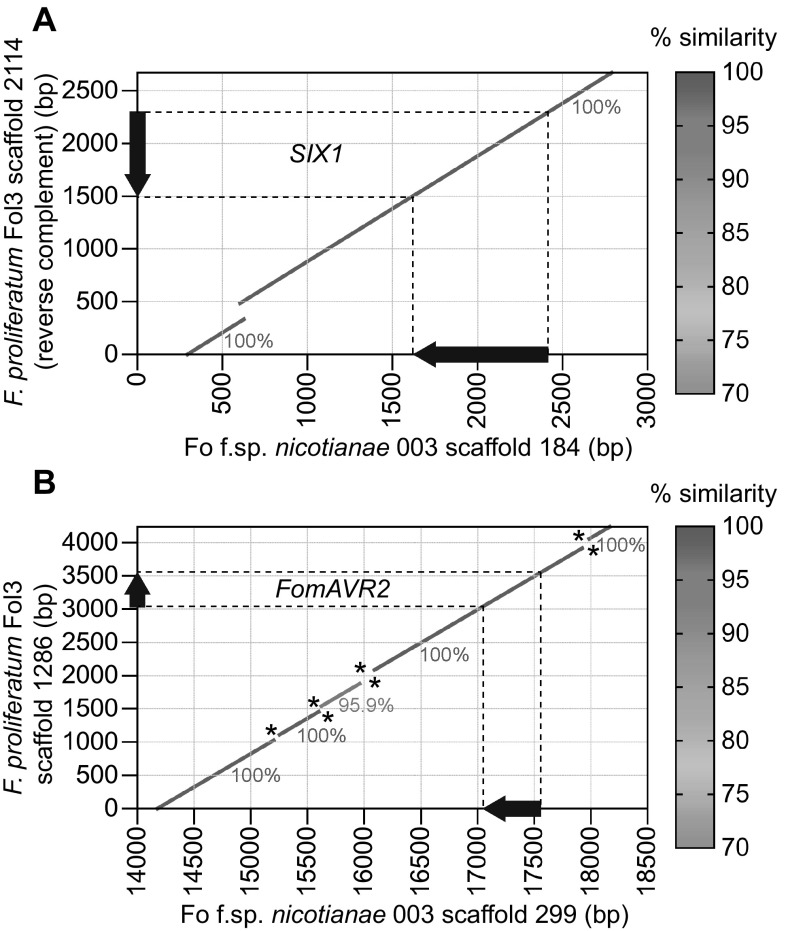
**a** *SIX1* (pseudogenized) and **b** *FomAVR2* are located within regions of 100% nucleotide sequence identity between Fol3 and Fonic003. Alignments were made with nucmer (with ‘-breaklen’ 100 to break alignments separated by *N*-gaps resulting from contig scaffolding). An *asterisk* positioned above the diagonal indicates an *N*-gap in Fol3 and an *asterisk* below the diagonal indicates an *N*-gap in Fonic003. The position of the *SIX1* and *FomAVR2* ORFs is indicated on the axes with a *black arrow*. For visualization reasons, the *x*-axis is only partially displayed; Fonic scaffold 184 is 11,173 bp and scaffold 299 is 20,768 bp long

Comparison of the scaffolds on which *SIX1* and *FomAVR2* are located in *F. proliferatum* Fol3 and Fo f. sp. *nicotianae* 003 by nucmer (MUMmer3) alignment shows that in both cases a 2 kb region containing the ORFs is 100% identical between these strains (Fig. 5). Multiple *N*-gaps are present in both assemblies, indicating the presence of many repetitive, difficult to assemble sequences (potentially transposons). Nonetheless, the successfully assembled stretches of sequence between these gaps are highly conserved between the two investigated strains, with only one pairwise alignment of 340 bp having a sequence identity score of less than 100% (95.9%). In the rest of the alignments no nucleotide differences were found between Fol3 and Fonic003. This level of sequence conservation is not expected between *F. oxysporum* and *F. proliferatum* assuming vertical inheritance (average sequence identity in conserved genes between Foniv015 and Hy14 is 96.1%).

### Vertical Inheritance of *SIX2*

**Fig. 6:**
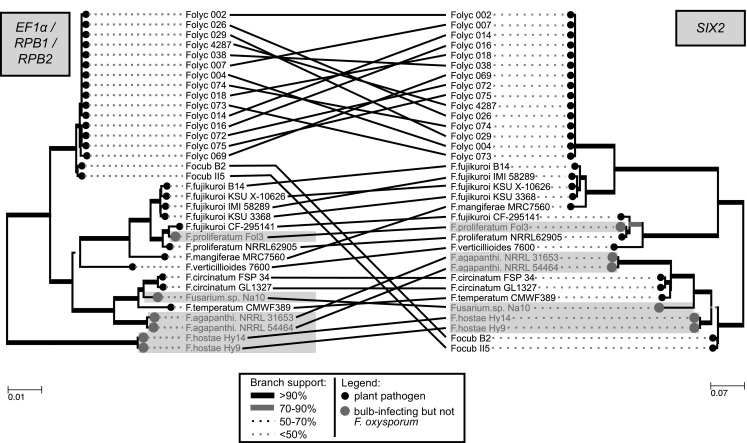
A tanglegram showing that the phylogeny of *SIX2* is largely congruent with the core phylogeny of *Fusarium* species that have a *SIX2* homolog. *SIX2* occurs in Fo f. sp. *lycopersici*, Fo f. sp. *cubense, F. hostae,* and numerous *Fusarium* species belonging to the FFSC. Nucleotide sequences were aligned with ClustalO and phylogeny was inferred using PhyML with 100 bootstraps (Focub: Fo f. sp. *cubense*; Folyc: Fo f. sp. *lycopersici*)

Within the FOSC, *SIX2* has only been identified in ff. spp. *lycopersici* and *cubense*. This gene, unlike the other 13 *SIX* genes, is very widespread throughout the *Fusarium fujikuroi species complex* (FFSC). Next to the two *F. agapanthi* strains*, F. proliferatum* Fol3*, F. hostae* Hy9/Hy14, and *Fusarium.* sp. Na10 that are mentioned in Table 1, a *SIX2* homolog was also identified in *F. circinatum* FSP34, *F. circinatum* GL1327, *F. fujikuroi* B14, *F. fujikuroi* IMI58289, *F. fujikuroi* KSU3368, *F. fujikuroi* KSUX-10626, *F. mangiferae* MRC7560, *F. temperatum* CMWF389, *F. verticillioides* 7600, *F. fujikuroi* CF-295141, and another *F. proliferatum* strain in our dataset: NRRL62905 (Fig. 6). All open reading frames were intact. The *SIX2* homologs in *F. proliferatum* strains Fol3 and NRRL62905 are identical. Moreover, comparison of the phylogenetic distribution of *SIX2* to the core phylogeny (based on the concatenated sequence alignment of *EF1alpha, RPB1,* and part of *RPB2*) shows that these trees are largely congruent with each other. Fo f. sp. *cubense* II5 and B2 form a notable exception to this, since they have a *SIX2* homolog that is only 69.4% identical to *SIX2* in Fo f. sp. *lycopersici* and that clusters closer to *SIX2* homologs in other *Fusarium* species (Fig. 6).

## Discussion

In this study, we performed genome-wide comparative analysis of *mimp* elements and effector genes in the *Fusarium* genus. *Mimps* have so far only been described in *F. oxysporum* and are contextually associated with virulence-associated genes in this species (Schmidt et al. 2013). Within *F. oxysporum*, *mimps* occur most frequently in the accessory chromosomes of plant-pathogenic strains (Bergemann et al. 2008; Schmidt et al. 2013). Bergemann et al. found that the enrichment in Fol4287 occurs only on “pathogenicity” chromosome 14 but not on chromosomes 3 and 6, which are also considered accessory but consist mostly of large segmental duplications. In another study, Dufresne et al. (2007) demonstrated that *mimp1* could be mobilized through the action of the transposase of *impalaE*, a *Tc1*-like autonomous element.

By searching for the conserved inverted repeat sequences that have been described for six families of *mimps* in 83 *F. oxysporum* and 52 other *Fusarium* genomes, we were able to extract 2572 *F. oxysporum mimps* and 116 *mimps* from other *Fusaria*. The relatively high abundance within the FOSC suggests that *mimps* originate from within this species complex. Six other *Fusarium* strains drew particular attention because relatively high numbers of *mimps* could be identified in their genomes. All of these strains were isolated from diseased flower bulbs.

Comparison of the nucleotide sequence of each *mimp* encountered in these strains showed that Fol3 *(F. proliferatum)*, Hy9, and Hy14 (both *F. hostae*) all have three to five elements in their genome that are 100% identical to *F. oxysporum mimps* (Fig. 3). This, combined with the fact that they may have an intact *impala* ORF (Fig. 2; assemblies are interrupted by N-gap or end of contig) and intact *SIX* gene homologs (other than *SIX2*) with high sequence similarity (Table 1) suggests that these isolates participated in horizontal chromosome transfer between *Fusarium* species.

In contrast to strains Fol3, Hy9, and Hy14 described above, the two *F. agapanthi* isolates (isolated from African lily in Australia and Italy) displayed a distinctly different *mimp* distribution. Most of the elements identified in these strains displayed long branch lengths and separate branching (Fig. 3), indicating a high level of sequence divergence from *F. oxysporum mimps*. *Fusarium* sp. Na10, isolated from *Narcissus* sp., only has four *mimps* in its genome assembly. Although this is a relatively high number for a strain belonging to the FFSC, all of the *mimp* sequences were different from Fo *mimps*.

Presence of *SIX* genes outside of the FOSC has so far been described in *Leptosphaeria maculans*, which has a distant homolog of *SIX1* [LmCys1, 26% amino acid identity (van de Wouw et al. 2010; Martin and Kamoun 2011)], *Colletotrichum orbiculare* and *C. higginsianum*, which have *SIX1* and *SIX6* homologs (Kleemann et al. 2012; Gan et al. 2013), *F. verticillioides*, which has a *SIX2* homolog (van der Does and Rep 2007) and NRRL31046, an isolate of *F. foetens* that has a (partial) *SIX1* homolog (Laurence et al. 2015). Interestingly, NRRL31046 was also isolated from a bulb flower, in this case from *Begonia* sp. displaying discoloration of veins in leaves and stems (Schroers et al. 2004). No genome sequence of this strain is currently available, and it would be interesting to further investigate the distribution of *SIX* genes amongst strains of *F. foetens* since this species also belongs to the FFSC.

We now find that *SIX2,* apart from being present in *F. verticillioides,* is widely distributed amongst species in the FFSC. The phylogeny of this gene is largely congruent with the species tree indicative of vertical inheritance (Fig. 6). This is not the case, however, for most other effector genes that were identified in the analysis including *SIX1, SIX6, SIX7*, and *FomAVR2* that seem to be largely restricted within the FOSC. The identification of *FomAVR2* and a pseudogenized copy of *SIX1*, both 100% identical to sequences in *F. oxysporum*, is suggestive of horizontal transfer to or from *F. proliferatum* Fol3. This hypothesis is strengthened by the sequences surrounding these genes (multiple kb in length) that are also 100% identical between the strains (Fig. 6). Additionally, the placement of *F. hostae* Hy9/Hy14 *SIX6* amongst the *F. oxysporum SIX6* homologs also points at a HT event of this gene, supported by the high bootstrap values of the *F. hostae* clade and high sequence similarity to Fo f. sp. *niveum* 015/019 *SIX6* (Fig. 4).

Horizontal chromosome transfer (HCT) within the *species complex* and across species boundaries is believed to have contributed to genetic diversity and the generation of new (pathogenic) variants (Ma et al. 2013; Kang et al. 2014). This has been experimentally shown so far only between pathogenic and non-pathogenic strains of *F. oxysporum* (Ma et al. 2010; Vlaardingerbroek et al. 2016; van Dam et al. submitted). In these studies, the pathogenicity chromosome of Fo f. sp. *lycopersici* (chr 14) or Fo f. sp. *radicis*-*cucumerinum* (chr^RC^) was transferred into the genetic background of biocontrol strain Fo47. Fo47 belongs to a different vegetative compatibility group than either of these pathogens. The recipient strain had subsequently become pathogenic towards tomato or several cucurbit species, respectively. The proposed mechanism for HCT is nuclear fusion followed by selective loss of chromosomes from one of the fusion partners, but exactly how this happens remains elusive (Vlaardingerbroek et al. 2016). Hyphal fusion of genetically dissimilar strains normally leads to a vegetative incompatibility response followed by programmed cell death (Glass and Dementhon 2006).

HT of virulence genes has been suggested for Fo f. sp. *canariensis* (Laurence et al. 2015) and also in strains of Fo that were isolated from natural ecosystems of Australia (Rocha et al. 2015). Interspecies HT of the virulence gene pisatin demethylase *(PDA)* from *Nectria haematococca* (the teleomorph of *F. solani*) to Fo f. sp. *phaseoli* and *pisi* was suggested by discordance between the gene genealogy of *PDA* and the organismal phylogeny (Milani et al. 2012). The presence of dispensable chromosomes in *N. haematococca* and *F. oxysporum* and the fact that these can move between strains in the FOSC led the authors of this study to suggest this as a potential pathway for horizontal transmission of chromosomes containing virulence genes between *Fusarium* species.

The alternative to HT, a shared origin of all *SIX* genes in the ancestor of the FOSC and FFSC followed by selective loss in most of the FFSC species remains a possibility. Since *SIX2* is found in multiple FFSC species as well as Fo, a shared origin at least for this gene is probable. For *SIX6, SIX7, SIX11*, and the Fo f. sp. *melonis* effector candidates, no homologs in other *Fusarium* species have been described until now (Schmidt et al. 2013, 2016). The compartmentalization of pathogen genomes (including that of Fo) and the concentration of effector genes on one or a few accessory chromosomes facilitates the simultaneous loss of multiple effector genes. The rate of loss of such an ancestral accessory chromosome could be high due to avirulence effects of the genes encoded on it. Still, it is hard to explain the high identity of some *mimps* and effector genes (and their up- and downstream regions) as the placement of non-Fo effector genes within the Fo clade under the assumption of exclusive vertical inheritance.

In conclusion, we describe here for the first time data suggestive of horizontal gene transfer between different *Fusarium* species. HT of (part of) an accessory chromosome may have occurred under natural conditions, such as in flower bulb fields. Data supportive of this hypothesis include the presence of many *mimps* (sometimes identical to a Fo *mimp*), as well as the occurrence of *SIX* homologs (other than *SIX2)* that were not found in other *Fusarium* species outside of the FOSC. Whole genome sequencing with long-read sequencing technologies of the *F. proliferatum* and *F. hostae* strains described here, Fo f. sp. *lilii*/*hyacinthi* as well as *F. foetens* NRRL31046 would be highly interesting in order to compare their genome architectures.

## Materials and Methods

### Identification of *mimps* and *impala* ORFs in each Genome

*Mimps* were identified using a custom python script (available upon request) that searches for the terminal inverted repeats using the following 16-nucleotide regular expression: ‘NNCAGT[GA][GA]G[GAT][TGC]GCAA[TAG]AA’. Stretches of sequence where an instance of this motif was found within 400 bp of another instance in reverse orientation were extracted. The list of newly identified *mimps* was then compared to each other through reciprocal BLASTN (*e* value < 1e−5, percent identity >80%, alignment length >160 nt) and clusters were formed with single linkage. This resulted in 40 clusters, of which the clusters with 10 or less *mimp* instances were grouped together in category ‘15’ (other). The sequences of *mimp* families 1–6 were extracted from Schmidt et al. (2013) and Bergemann et al. (2008) and compared to the clusters obtained from reciprocal BLAST to see which of the categories represented which family.

Detection of an intact impala ORF was performed by manual evaluation of TBLASTN output (*e* value < 1e−100) using the full-length FOM24 *impala* transposase ORF (Genbank accession AF282722.1) as a query.

### Multiple Sequence Alignments and Phylogeny

For alignment of conserved genes, the sequences of *EF1α, RPB1*, and *RPB2* were extracted from the genomes based on BLASTN searches, using the gene sequences of Fol4287 as query. ClustalO v1.2.1 (Sievers et al. 2014) was used to make an alignment for each gene, after which the alignments were concatenated into a single alignment. This concatenated alignment was trimmed using TrimAl (with -strictplus) (Capella-Gutierrez et al. 2009) and fed to PhyML v20120412 (with -bootstrap 100) (Guindon et al. 2009) in order to retrieve a phylogeny of the *Fusarium* genomes. A similar approach was taken for identification and alignment of *SIX* and Fo f. sp. *melonis* effector candidate homologs, although these alignments were not trimmed.

MAFFT v6.903b (Katoh et al. 2002) was used for alignment of *mimp* sequences and PhyML was applied for phylogenetic inference as described above. All trees were visualized in ETE3 v3.0.0b35 (Huerta-Cepas et al. 2016).

### Whole Genome Sequencing, De Novo Assembly

*Fusarium* genomic DNA was isolated through phenol–chloroform extraction from freeze-dried mycelium that was harvested from 5-day old NO_3_-medium (0.17% yeast nitrogen base, 3% sucrose, 100 mM KNO_3_) cultures as described in detail in van Dam et al. (2016). Library preparation of insert size 550 bp and Illumina HiSeq 2500 paired-end sequencing was performed at Keygene N. V. (Wageningen, the Netherlands).

Sequencing reads were trimmed for quality and to remove adapter sequences with FastqMcf v1.04.676 (https://expressionanalysis.github.io/ea-utils/, quality threshold = 20). De novo assemblies were generated using CLC-workbench 8.0. Default settings were used, except ‘minimum contig length = 500’.

### Alignment of Scaffolds

Nucmer of the MUMmer package v3.1 (with ‘-breaklen 100’ to break alignments separated by *N*-gaps resulting from contig scaffolding) was used for visualization of scaffold alignments.

### Data Access

Whole-Genome Shotgun projects for the newly sequenced strains of *F. hostae* Hy9, Hy14, *F. proliferatum* Fol3, and *Fusarium* sp. Na10 have been deposited at Genbank under the BioProject PRJNA389502. Raw sequence data have been deposited into the Sequence Read Archive under the accession number SRP109077. All publically available genome sequences that were used were obtained from Genbank. Their NCBI accession numbers can be found in Supplementary Table S1.

## Electronic supplementary material

Below is the link to the electronic supplementary material.

Supplementary material 1 (XLSX 19 kb) Table S1: Number of *mimps* identified in each of the genome assemblies used in this study and the GenBank accession numbers of each assembly (accessed on 10-4-2017)
